# LIN‐28 balances longevity and germline stem cell number in *Caenorhabditis elegans* through let‐7*/*AKT*/*DAF‐16 axis

**DOI:** 10.1111/acel.12539

**Published:** 2016-10-11

**Authors:** Dan Wang, Lei Hou, Shuhei Nakamura, Ming Su, Fang Li, Weiyang Chen, Yizhen Yan, Christopher D. Green, Di Chen, Hong Zhang, Adam Antebi, Jing‐Dong J. Han

**Affiliations:** ^1^ Key Laboratory of Computational BiologyCAS Center for Excellence in Molecular Cell ScienceCollaborative Innovation Center for Genetics and Developmental BiologyChinese Academy of Sciences‐Max Planck Partner Institute for Computational BiologyShanghai Institutes for Biological SciencesChinese Academy of Sciences320 Yue Yang RoadShanghai200031China; ^2^ University of Chinese Academy of ScienceBeijing100049China; ^3^ Max Planck Institute for Biology of AgeingJoseph‐Stelzmann‐Strasse 9bCologne50931Germany; ^4^ State Key Laboratory of Pharmaceutical Biotechnology and MOE Key Laboratory of Model Animals for Disease StudyModel Animal Research CenterNanjing UniversityNanjingJiangsu210061China; ^5^ Institute of BiophysicsChinese Academy of SciencesBeijing100101China

**Keywords:** *Caenorhabditis elegans*, DAF‐16, let‐7, LIN‐28, longevity, reproduction

## Abstract

The RNA‐binding protein LIN‐28 was first found to control developmental timing in *Caenorhabditis elegans*. Later, it was found to play important roles in pluripotency, metabolism, and cancer in mammals. Here we report that a low dosage of *lin‐28* enhanced stress tolerance and longevity, and reduced germline stem/progenitor cell number in *C. elegans*. The germline LIN‐28*‐*regulated microRNA let‐7 was required for these effects by targeting *akt‐1/2* and decreasing their protein levels. AKT‐1/2 and the downstream DAF‐16 transcription factor were both required for the lifespan and germline stem cell effects of *lin‐28*. The pathway also mediated dietary restriction induced lifespan extension and reduction in germline stem cell number. Thus, the LIN‐28/let‐7/AKT/DAF‐16 axis we delineated here is a program that plays an important role in balancing reproduction and somatic maintenance and their response to the environmental energy level—a central dogma of the ‘evolutionary optimization’ of resource allocation that modulates aging.

## Introduction

Aging can be caused by a limited capacity of somatic maintenance and repair. Germline stem cells, as the only immortal cells in an organism, need elevated levels of maintenance and repair. As an organism and a species, the plastic ‘evolutionary optimization’ process has shaped an intricate balance between reproduction (or germline progenitor pool size) vs. aging (or somatic maintenance and repair) under limited resources and energy availability to the organism (Kirkwood, 2005). However, it is unclear whether there exists a genetic program that determines this balance.

LIN‐28, an RNA‐binding protein, was first identified in *Caenorhabditis elegans* through screening for genes that alter developmental timing (Arasu *et al*., 1991). Mutations in *lin‐28* cause precocious development, where many developmental events specific to the second larval stage (L2 stage) are skipped and later events occur one or two stages earlier than normal (Moss *et al*., 1997). Consistent with its role in controlling L2‐specific cell fate, *lin‐28* is highly expressed in the embryo, L1 and L2 stages, and decreases in L3 stage, which leads to the accumulation of microRNA let‐7. *lin‐28* shows a very weak expression in young adult *C. elegans* (Reinhart *et al*., 2000; Van Wynsberghe *et al*., 2011). However, the expression pattern of *lin‐28* during aging has not been studied. By RNA‐seq and PCR of dissected gonads, Jungkamp *et al*. confirmed germline expression of *lin‐28* mRNA in young adult worms, implicating that LIN‐28 may play roles in adult worms in addition to developmental timing (Jungkamp *et al*., 2011).

By directly binding to pri‐let‐7, LIN‐28 blocks the maturation of let‐7, whereas let‐7 targets LIN‐28 and represses its translation (Newman *et al*., 2008). This bi‐stable switch regulates biological processes such as stem cell self‐renewal, glucose metabolism, development, and cancer (Shyh‐Chang & Daley, 2013). When overexpressed in mice, both Lin28a and Lin28b promote an insulin‐sensitized state that resists high‐fat diet induced diabetes, partially through let‐7 (Zhu *et al*., 2011). Lin28a reactivation also enhanced mouse tissue repair by binding and enhancing the translation of mRNAs for several metabolic enzymes, leading to increased glycolysis and oxidative phosphorylation. In this case, Lin28‐induced repression of let‐7 was necessary but insufficient to account for the effects of Lin28 (Shyh‐Chang *et al*., 2013). Shinoda *et al*. have found that the Lin28a/let‐7 axis also plays a role in mouse germ lineage determination. Compared to wild‐type, *Lin28a* knockout mice have a reduced size of the germ cell pool during embryogenesis, leading to impaired fertility in both male and female adults and overexpression of *let‐7* also reduces the germ cell pool (Shinoda *et al*., 2013).

Several heterochronic factors have been shown to regulate aging in *C. elegans*, among them, *lin‐4* and its target LIN‐14 was the first pair discovered. Loss of function of the microRNA *lin‐4* shortens lifespan, while mutating its target gene, *lin‐14* extends lifespan. Both *lin‐4* and *lin‐14* require the downstream effector DAF‐16 to influence lifespan (Boehm & Slack, 2005). DAF‐12, a steroid receptor, which is involved in the L2–L3 transition as well as its transcriptional target miR‐84 and miR‐241 regulate aging through the germline (Shen *et al*., 2012). Additionally, loss of function of SEA‐2, a zinc‐finger protein that regulates developmental timing in *C. elegans,* promotes longevity in a DAF‐16‐dependent manner (Huang & Zhang, 2011).

Despite the well‐known functions of LIN‐28 in development, stem cell maintenance, metabolism, and cancer, little is known about its role in aging and lifespan control. Studies have shown that LIN28 regulates insulin sensitization and germ cell pool size in mice (Zhu *et al*., 2011; Shinoda *et al*., 2013). Given the fact that both insulin pathway and reproduction contribute to aging, we hypothesized that LIN28 may regulate the aging process. Here we show that knockdown of *lin‐28* extends lifespan and promotes meiotic entry of germline stem cells in the model organism *C. elegans*. Loss of *lin‐28* leads to a much smaller number of germline stem cells in young adult worms. The lifespan effect of *lin‐28* is dependent on an intact germline, as *lin‐28* RNAi cannot extend the lifespan of *glp‐1* mutant worms. As the most well‐known downstream effector of LIN‐28, let‐7 is indispensable for LIN‐28 induced longevity and smaller germline progenitor pool. By targeting AKT‐1/2, let‐7 stimulates translocation of DAF‐16. Germline stem cell and lifespan effects of *lin‐28* RNAi are both abolished in *let‐7*,* akt‐1*,* akt‐2,* and *daf‐16* mutant worms, indicating that the LIN‐28/let‐7/AKT/DAF‐16 axis is a program that plays an important role in balancing reproduction and somatic maintenance.

## Results

### Knockdown of *lin‐28* extends *C. elegans* lifespan

**Figure 1:**
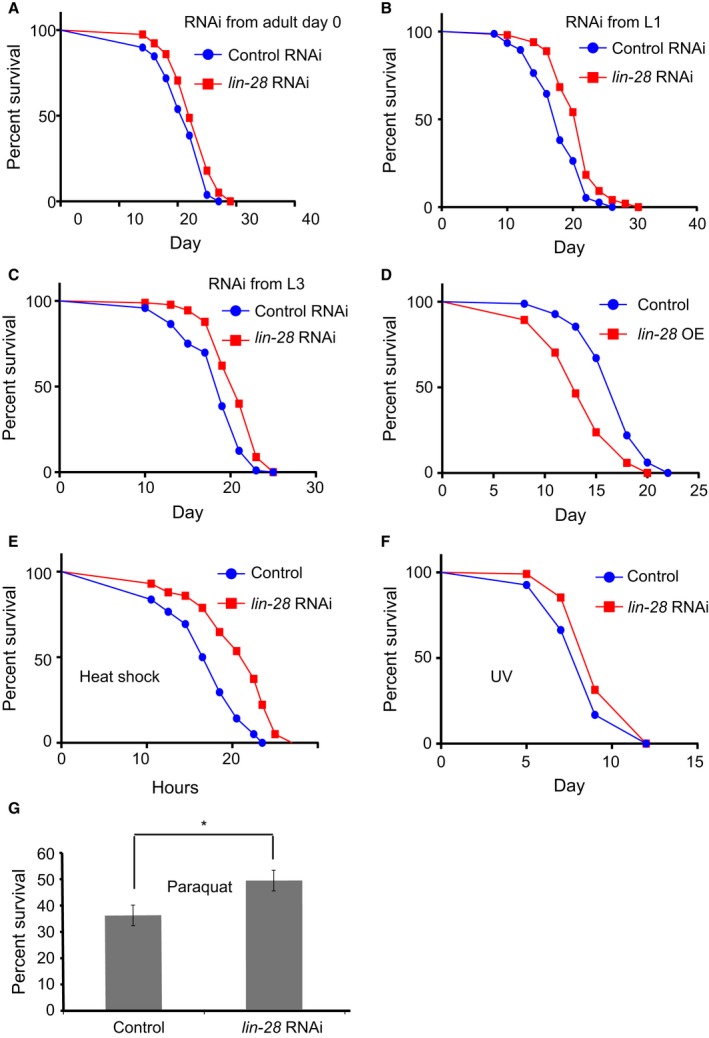
*lin‐28* knockdown extends *C. elegans* lifespan and enhances stress tolerance. (A) *lin‐28* RNAi initiated from adulthood extends the mean lifespan by 8.6% (log‐rank test *P* = 0.0016). (B) RNAi of *lin‐28* initiated from L1 stage extends the mean lifespan of wild‐type worms by 20.3% (log‐rank test *P* < 0.0001). (C) *lin‐28* RNAi initiated from L3 stage extends the mean lifespan of wild‐type worms by 16.9% (log‐rank test *P* < 0.0001). (D) Worms overexpressing *lin‐28* have an average of 17.2% shorter lifespan compared to wild‐type (log‐rank test *P* < 0.0001). (E) When shifted to 35 °C on adult day 3, *lin‐28* RNAi worms survive better than wild‐type worms (log‐rank test *P* < 0.0001). (F) For DNA damage treatment, worms were exposed to 2000 KJ m^−2^ UV light and survival rates were assayed every other day. Worms treated with *lin‐28* RNAi survive better than wild‐type worms after UV treatment (log‐rank test *P* = 0.0008). (G) For oxidative stress treatment, worms were immersed in 300 mM paraquat on adult day 3. Survival rates were assayed after 12 h of treatment. Worms treated with *lin‐28* RNAi show a 48.9% survival rate, while the controls with empty vector have a 36% survival rate (two‐tailed Student's *t*‐test *P* = 0.013). *P < 0.05. Quantitative data and statistical analyses are included in Table S1(Supporting information). See also Fig. S1 and Table S1 (Supporting information).

When worms are fed with bacteria containing double‐stranded RNA against *lin‐28* from young adult and onwards, they showed an 8.6% extension of lifespan compared to those fed with empty vector bacteria as control (Fig. 1A, Table S1). Starting *lin‐28* RNAi from L1 larval stage, instead of young adult, had a stronger lifespan extension effect (20.3% extension, Fig. 1B, Table S1), and this stronger lifespan extension was not caused by different RNAi efficiencies (Fig. S1A). To rule out the possibility that this longer lifespan is caused by the heterochronic effect of LIN‐28, we also fed worms *lin‐28* RNAi bacteria from the beginning of L3 stage, right after L2 stage, at which LIN‐28 mainly functions to regulate seam cell fate. We found that *lin‐28* RNAi from L3 stage extended lifespan to a similar extent as from L1 stage and still longer than that of RNAi from adult stage (Fig. 1C, Table S1). From these results, we concluded that the lifespan extension effect of LIN‐28 can be separated from its heterochronic effect, whereas the L3 and L4 stages, at which germline stem cell/progenitor pool quickly expands, are critical for lifespan regulation by LIN‐28.

Consistent with the longer lifespan extension by *lin‐28* knockdown, the *lin‐28* overexpression strain, which overexpresses *lin‐28* mRNA by threefold to fivefold (Fig. S1B), had a much shorter lifespan (17.2% reduction) compared to wild‐type worms (Fig. 1D, Table S1), further confirming the role of LIN‐28 in lifespan regulation.

Other than a longer lifespan, worms with low level *lin‐28* expression also showed smaller body sizes and a much lower fat content, as indicted by oil‐red intensity, compared to worms fed with empty vector bacteria as control (Fig. S1C and S1D).

### LIN‐28 modulates heat, UV, and oxidative stress responses

Given the close association between lifespan regulation and stress tolerance, we further examined whether *lin‐28* affects stress tolerance in *C. elegans*. Here, we used 35 °C heat shock, UV, and paraquat treatments to represent heat, DNA damage, and oxidative stresses, respectively. Consistent with the lifespan extension by *lin‐28* knockdown, *lin‐28* RNAi conferred stronger resistance in worms to all three stresses to various extents (Fig. 1E–G).

### LIN‐28 is required for proper establishment of the germline progenitor pool

**Figure 2:**
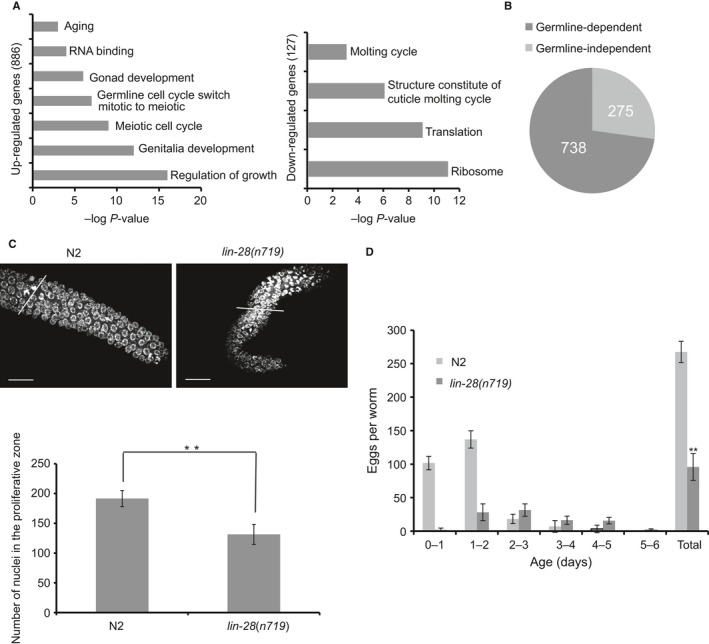
LIN‐28 is required for proper establishment of the germline progenitor pool. (A) Top gene ontology (GO) terms enriched among genes up‐ or downregulated by *lin‐28* RNAi. (B) RNA‐seq analysis identifies a total of 1013 differentially expressed genes (DEGs) in *lin‐28* RNAi worms vs. control empty vector fed worms; while in the *glp‐1* worms which lack the germline, it identifies only 275 DEGs, indicating that most DEGs induced by low *lin‐28* are germline dependent. (C) Representative DAPI‐stained and average number of proliferative zone nuclei in early adult wild‐type and *lin‐28(n719)* mutant worms. Scale bars, 20 μm. **P < 0.01 by two‐tailed Student's *t*‐test. See Table S2 (Supporting information) for complete data. (D) Number of progenies produced by wild‐type and *lin‐28*(*tm4566*) mutant worms. **P < 0.01 by two‐tailed Student's *t*‐test. See also Fig. S2, Tables S2 and S4 (Supporting information).

Consistent with a published result (Jungkamp *et al*., 2011), we found by qRT–PCR that in adult worms *lin‐28* mainly expressed in the germline (Fig. S2A). To identify LIN‐28‐regulated genes, we performed a genomewide RNA‐seq analysis at adult day 4 for worms fed with *lin‐28* RNAi or empty vector bacteria from L1 stage. We found that compared to the control worms, 886 genes are upregulated and 127 are downregulated by *lin‐28* RNAi (Fig. 2A, Table S4). In addition to ‘regulation of growth’, the top gene ontology (GO) enrichment categories for these genes include ‘meiotic cell cycle’ and ‘germline cell cycle switch, mitotic to meiotic’ (Fig. 2A). As the germline is the only tissue in *C. elegans* in which cell division continues to occur into adulthood, we hypothesized that LIN‐28 mainly works through the germline to influence lifespan. We therefore conducted RNA‐seq of *lin‐28* knockdown by RNAi in *glp‐1(e2141)* mutant worms, which develop only 5–15 meiotic germ cells and thus lack germline when shifted to the restrictive temperature at late L1 stage. We found that the majority (73%) of LIN‐28‐regulated genes are germline dependent, as their transcript levels are affected by *lin‐28* RNAi in wild‐type worms but not in *glp‐1*(*e2141*) mutant worms (Fig. 2B). Interestingly, we found that knockdown of *lin‐28* shares many downstream genes with that of *glp‐1* loss (Fisher's exact test *P* = 1.0 × 10^−16^) and these overlapped genes are functionally enriched in ‘meiotic cell cycle’ (Fig. S2B).

Then, we counted proliferative germ cells of the *lin‐28*(*n719*) mutant. Similar to the phenotype of fewer nuclei in the proliferative zone characterized by *glp‐1(e2141)* loss, the *lin‐28* mutant worms had a significantly reduced number of germline progenitor cells (Fig. 2C, Table S2) and much smaller brood size than wild‐type (*P* = 9.4 × 10^−13^ by *t*‐test) (Fig. 2D).

Next, we investigated whether the decrease of germline progenitor cell number in *lin‐28* mutant is due to decreased cell proliferation (cell cycle defect) or increased differentiation (disrupted balance between proliferation and differentiation). To determine whether the frequency of germ cell division was altered in *lin‐28* mutant, we measured the mitotic index, the percentage of metaphase and anaphase nuclei over the total number of nuclei in the proliferative zone. In *lin‐28* mutant worms, mitotic index was not changed significantly, indicating that LIN‐28 does not influence cell cycle (Fig. S2C). The distance from the distal tip to the transition zone is a measure of the effective reach of the DTC signal to deter meiotic entry (Michaelson *et al*., 2010). We examined this parameter in *lin‐28* mutant and found that the position of meiotic entry was shifted distally in *lin‐28* mutant, indicating that LIN‐28 affects the balance between mitotic (undifferentiated) and meiotic (differentiated) state (Fig. S2D). This phenotype is highly consistent with the enrichment for the ‘germline cell cycle switch, mitotic to meiotic’ function in *lin‐28* RNAi induced differentially expressed genes (Fig. 2A).

### Lin‐28 acts germline dependently to regulate germline progenitor pool size and longevity

**Figure 3:**
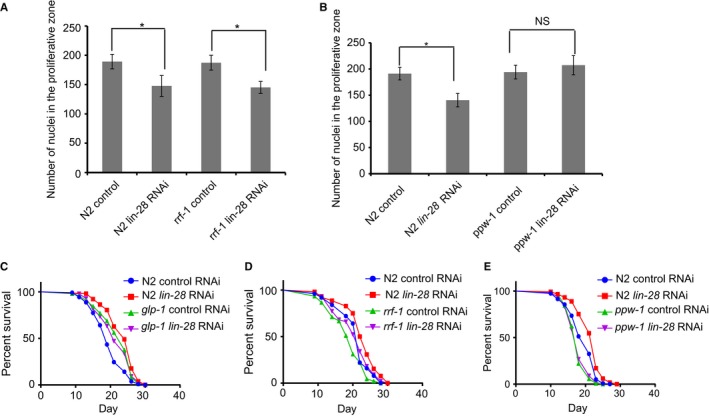
Germline *lin‐28* is required for germline stem cell number and lifespan regulation. (A) Average number of proliferative zone nuclei in germlines of early adult worms, wild‐type, *rrf‐1*(*pk1417*) with control and *lin‐28* RNAi. * *P* < 0.05 by two‐tailed Student's *t*‐test. (B) Average number of proliferative zone nuclei in germlines of early adult worms, wild‐type, *ppw‐1*(*pk2505*) with control and *lin‐28* RNAi. *P < 0.05 by two‐tailed Student's *t*‐test. NS, not significant. (C) In *glp‐1* (*e2141*) worms lacking an intact germline, *lin‐28* RNAi does not extend lifespan (log‐rank test *P* = 0.44). (D) In *rrf‐1(pk1417)* mutant worms, where RNAi only works in germline, *lin‐28* RNAi still extends lifespan to a similar extent as in N2 (log‐rank test *P* < 0.0001). (E) In *ppw‐1*(*pk2505*) mutant worms, where RNAi only is efficient in soma, *lin‐28* RNAi cannot extend lifespan anymore (log‐rank test *P* = 0.50). See also Fig. S2, Tables S1 and S2 (Supporting information).

To compare the germline vs. somatic activity of LIN‐28 in regulating germline progenitor cell number, we used the *rrf‐1(pk1417)* mutant for which RNAi is predominantly effective only in germline with a minor effect in the intestine (Smardon *et al*., 2000; Kumsta & Hansen, 2012). We found that knockdown of *lin‐28* in germline showed a similar effect on the germline progenitor cell number to that of whole‐body knockdown of LIN‐28 (21.9% reduction in *rrf‐1* vs. 22.4% in wild‐type, Fig. 3A, equal *lin‐28* RNAi efficiency is observed in N2 and *rrf‐1* strains by q‐PCR, Figs S2E,F, Table S2) while *lin‐28* RNAi in the soma only RNAi strain, *ppw‐1*(*pk2505*), hardly reduced number of germline progenitor cells (Fig. 3B), suggesting that LIN‐28 mainly acts in the germline to regulate germline stem/progenitor cell number.

Next, we tested whether the lifespan effect of LIN‐28 is also germline dependent. Like the germline dependency of *lin‐28*'s effects on proliferative germline nuclei number, *lin‐28* RNAi could not extend lifespan of *glp‐1(e2141)* mutant worms which lack germline (Fig. 3C, Table S1). In the *rrf‐1*(*pk1417*) mutant strain, *lin‐28* RNAi still extended lifespan to a similar extent as in N2 worms (Fig. 3D, Table S1). In the *ppw‐1*(*pk250*5) mutant worms, where RNAi only works in the soma, *lin‐28* RNAi could not significantly extend lifespan (Fig. 3E, Table S1). Thus, LIN‐28 mainly works in the germline to regulate germline progenitor number and lifespan.

### LIN‐28 mediates effects of diet on germline progenitor pool size and lifespan regulation

**Figure 4:**
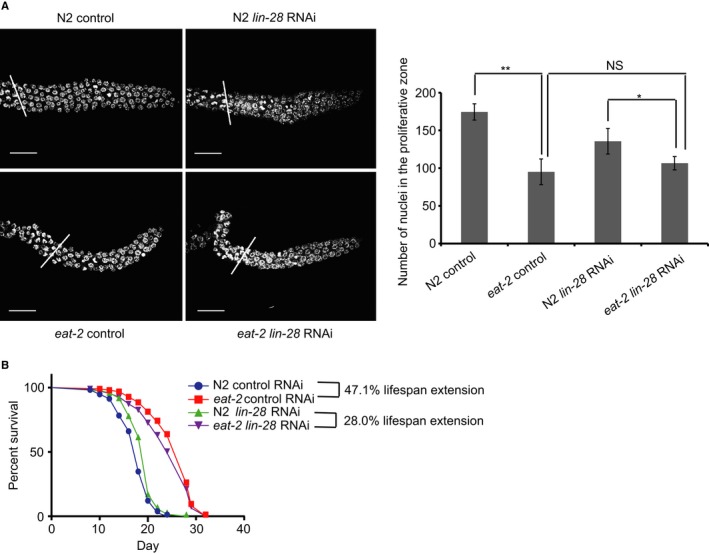
*lin‐28* responds to dietary restriction to influence germline stem cell proliferation and longevity. (A) Number of proliferative zone nuclei in *eat‐2*(*ad1116*) mutant worms fed with control bacteria and *lin‐28* RNAi bacteria. **P < 0.001, *P < 0.05. (B) *eat‐2(ad1116)* mutant extends median lifespan by 47.1% compared to wild‐type worms (log‐rank test *P* < 0.0001). *eat‐2(ad1116)* mutant extends lifespan by 28.0% compared to wild‐type worms when subjected to *lin‐28* RNAi (log‐rank test *P* < 0.0001). See also Fig. S3, Tables S1 and S2 (Supporting information).

In mice, Lin28 has been shown to play roles in glucose metabolism (Zhu *et al*., 2011) and glycolysis during tissue repair (Shyh‐Chang *et al*., 2013). To determine whether LIN‐28 links nutritional signals to germline progenitors in *C. elegans*, we used the *eat‐2*(*ad1116*) mutant as a genetic mimic of dietary restriction (DR). Consistent with the result of Korta *et al*. that dietary restriction strongly reduces the number of proliferative germ cells (Korta *et al*., 2012), *eat‐2* mutant has a much smaller germline stem cell number compared to N2 worms. Importantly, the extent of germline stem cell number reduction caused by *eat‐2* mutant was greatly attenuated by *lin‐28* knockdown (45.5% reduction in *eat‐2* control vs. N2 control; 21.4% reduction in *eat‐2 lin‐28* RNAi vs. N2 *lin‐28* RNAi, Fig. 4A, Table S2). Meanwhile, under *eat‐2* mutant genetic background, *lin‐28* RNAi cannot significantly reduce germline stem cell number (Fig. 4A, Table S2). We also examined the effect of DR treatment on the germline in the presence and absence of *lin‐28*. Dietary restriction was conducted by feeding worms with 1x10^8^ cfu mL^−1^ bacteria and compared to control worms fed *ad libitum* with 1x10^10 ^cfu mL^−1^ bacteria. We found that wild‐type worms under DR had 67.2% fewer progenitors than *ad libitum* siblings, while *lin‐28(n719)* mutants grown under DR contained only 14.8% fewer germline progenitors than *ad libitum* siblings (Fig. S3A, Table S2). This attenuated response of *lin‐28* RNAi/mutant worms to DR suggests a role for LIN‐28 in connecting nutrient sensing in the developmental stage to germline plasticity.

To examine whether *lin‐28* also mediates DR's effect on lifespan, we conducted lifespan assay of *eat‐2*(*ad1116*) mutant feeding with *lin‐28* RNAi bacteria. *eat‐2* mutation extended lifespan by 47.1% under control condition (*eat‐2* control vs N2 control, Fig. 4B, Table S1), while it only extended lifespan by 28% when subjected to *lin‐28* RNAi (*eat‐2 lin‐28* RNAi vs. N2 *lin‐28* RNAi, Fig. 4B, Table S1). Unlike in wild‐type worms, *lin‐28* RNAi cannot significantly extend lifespan of *eat‐2* mutant worms (*P* = 0.102) (Fig. 4B). To further confirm the response of LIN‐28 to diet in regulating longevity, we conducted lifespan assay of *lin‐28* RNAi worms under DR treatment as described above. In this DR experiment, to maintain the activity of *lin‐28* RNAi bacteria, the bacteria were not UV‐killed as usual, but left to grow on the plates when fed to worms, which might have partially compromised the DR effect on lifespan. When feeding worms with empty vector bacteria, DR extended lifespan by 7.1% (Fig. S3B, Table S1), but when *lin‐28* was knocked down by RNAi, DR cannot extended lifespan significantly (Fig. S3C, Table S1). This attenuated response of *lin‐28* RNAi worms to DR treatment also suggests a role for LIN‐28 in connecting nutrient sensing to lifespan regulation.

### Let‐7 is required for LIN‐28‐mediated longevity and germline stem/progenitor cell number

**Figure 5:**
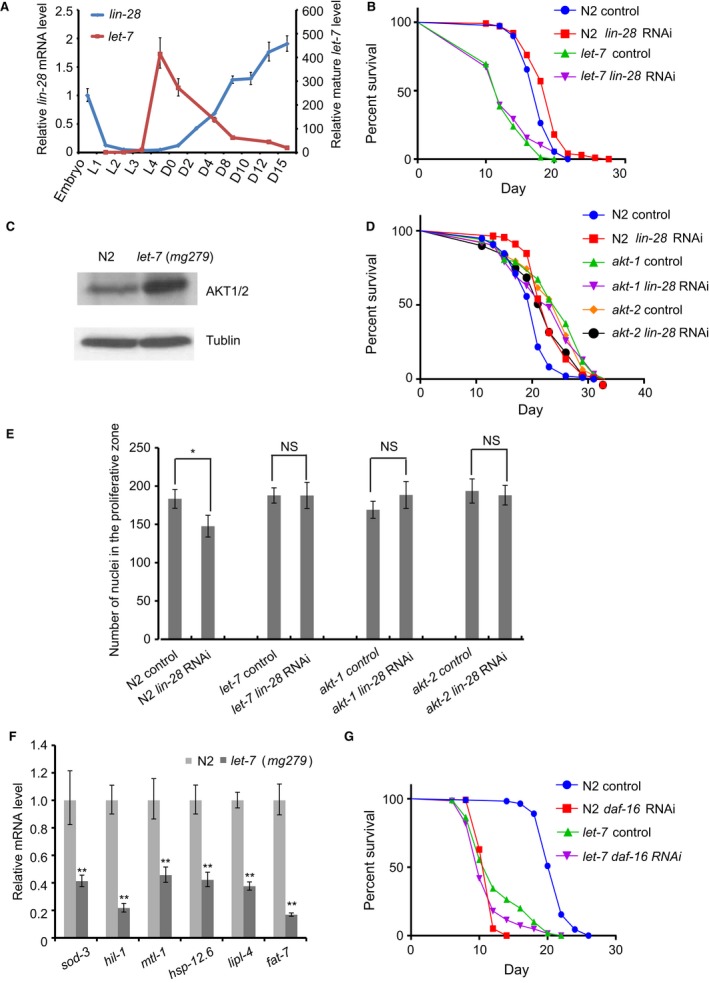
*lin‐28* exerts its effects on germline stem cell number and lifespan through let‐7 and AKT‐1/2. (A) Expression patterns of *lin‐28* and mature *let‐7* are anticorrelated through development and aging. The *lin‐28* mRNA decreases after L2 stage but increases during aging, while mature *let‐7* increases after L2 stage but decreases during aging. Data represent mean ± SD (n = 3 biological repeats). (B) *lin‐28* RNAi does not extend the lifespan of *let‐7*(*mg279*) mutant worms (log‐rank test *P* = 0.33). (C) Protein level of AKT‐1/2 is increased in *let‐7*(*mg279*) mutant worms (*P* < 0.0001 by *t*‐test based on 3 biological replicates). (D) *lin‐28* RNAi cannot extend the lifespan of *akt‐1*(*ok525*) and *akt‐2*(*ok393*) mutants (log‐rank *P* = 0.42 and *P* = 0.06, respectively). (E) *lin‐28* RNAi reduce germline stem cell number of wild‐type worms significantly, but does not change germline stem cell number of *let‐7*,* akt‐1,* and *akt‐2* mutant worms. *P < 0.05 by two‐tailed Student's *t*‐test. NS, not significant. (F) qRT–PCR quantification of mRNA for DAF‐16 target genes in the *let‐7*(*mg279*) mutant. Data represent mean ± SD (n = 3 biological repeats). **P < 0.0001. (G) RNAi of *daf‐16* slightly but insignificantly shortens lifespan of *let‐7*(*mg279*) mutant worms (log‐rank test *P* = 0.29) but not to the same extent as that for N2 (log‐rank test *P* < 0.0001). See also Fig. S4, Tables S1 and S2 (Supporting information).

The most well‐demonstrated function of LIN‐28 is to repress the maturation of pri‐let‐7. We measured the mature let‐7 expression during aging and found that the mature let‐7 RNA level is anticorrelated with the mRNA level of *lin‐28* at both developmental and aging stages. We found that the mature let‐7 level began to accumulate at L3 stage right after the sharp decrease of *lin‐28* and peaked at L4 stage. During adulthood, the *lin‐28* mRNA level increased, while mature *let‐7* decreased with age (Fig. 5A). Mature *let‐7* level increases in both *lin‐28* mutant and *glp‐1* mutant (Fig. S4A). As *let‐7* null allele is lethal which makes lifespan assay impossible, we used a weak allele of *let‐7*(*mg279*) which has ~9% residual mature let‐7 compared with wild‐type worms (Fig. S4A). This *let‐7* mutant worm showed a much shorter lifespan (Fig. 5B) and a larger brood size than wild‐type worms (Fig. S4B). *lin‐28* RNAi could not extend lifespan of this *let‐7* mutant (Fig. 5B, Table S1), which indicates that let‐7 lies downstream of LIN‐28 in regulating lifespan. Consistent with *lin‐28*'s role in DR response, *let‐7*(*mg279*) mutant partially suppressed DR induced longevity (Fig. S4C, Table S1). To search for the potential targets of let‐7*,* we used the miRWIP target prediction package (Hammell *et al*., 2008), which predicted both AKT‐1 and AKT‐2 as targets. let‐7 has the same seed region as miR‐84, miR‐241, and miR‐48 in *C. elegans*, and Shen *et al*. have demonstrated that AKT‐1 is one of the targets of miR‐84 and miR‐241 (Shen *et al*., 2012), we therefore compared the protein level of AKT1/2 by Western blot with an antibody that recognizes both AKT‐1 and AKT‐2 in *let‐7(mg279)* mutant worms and wild‐type N2 worms. Indeed, the AKT protein level increased in the *let‐7* mutant worms compared to the wild‐type control, which indicates that let‐7 also regulates AKTs (Fig. 5C). Consistent with this finding, *lin‐28* RNAi could not significantly extend lifespan of either *akt‐1(ok525)* mutant or *akt‐2(ok393)* mutant worms (Fig. 5D, Table S1), demonstrating that AKT‐1 and AKT‐2 act downstream of the LIN‐28/let‐7 axis in regulating lifespan.

Similar to the epistatic relationship of *lin‐28* with *let‐7* and *akt‐1/2* in regulating lifespan, *lin‐28* RNAi effect on germline stem/progenitor cell number was abolished in *let‐7* mutant and *akt‐1/2* mutant (Fig. 5E, Table S2). As AKT‐1 and AKT‐2 are well‐known upstream regulators of DAF‐16, we thus tested whether DAF‐16 activity are regulated by loss of *let‐7*. Using gene set enrichment analysis (GSEA), we found that DAF‐16‐targeted genes are significantly enriched in the genes downregulated by loss of *let‐7* (*P* = 1.6 × 10^−13^, Fig. S4D). Then, we selected several microarray‐identified or classical DAF‐16‐targeted genes for qPCR validation (Murphy, 2006). All six tested genes were downregulated in *let‐7* mutant worms (Fig. 5F). We further examined the epistasis relationship between *let‐7* and *daf‐16* in regulating longevity. We found that there was no significant difference between the lifespan of *let‐7* mutant and wild‐type worms when both are subjected to *daf‐16* RNAi (Fig. 5G, Table S1), suggesting that DAF‐16 acts downstream of the LIN‐28/let‐7 axis in regulating longevity.

As AKT‐1/2 and DAF‐16 are well‐known members of the IIS pathway and act downstream of DAF‐2, and in mice, IGF1R, the homolog of DAF‐2, is one of the direct targets of *let‐7*, we also examined the relationship between *daf‐2(e1370)* and *lin‐28*. Unlike *Igf1r* in mice, *daf‐2* has no conserved let‐7 binding sites in its 3’ UTR according to both of TargetScan and miRWIP predictions; thus, it is unlikely to be a direct target of let‐7 in worms. Consistent with these prediction results, both the *daf‐2* mutant fed with *lin‐28* RNAi bacteria and *lin‐28* overexpressed worms fed with *daf‐2* RNAi bacteria showed additive effects on lifespan, indicative of independent roles of the two alleles in regulating lifespan (Fig. S4E,F, Table S1).

### Loss of *daf‐16* abolishes LIN‐28's effect on longevity and germline stem/progenitor cell number

**Figure 6:**
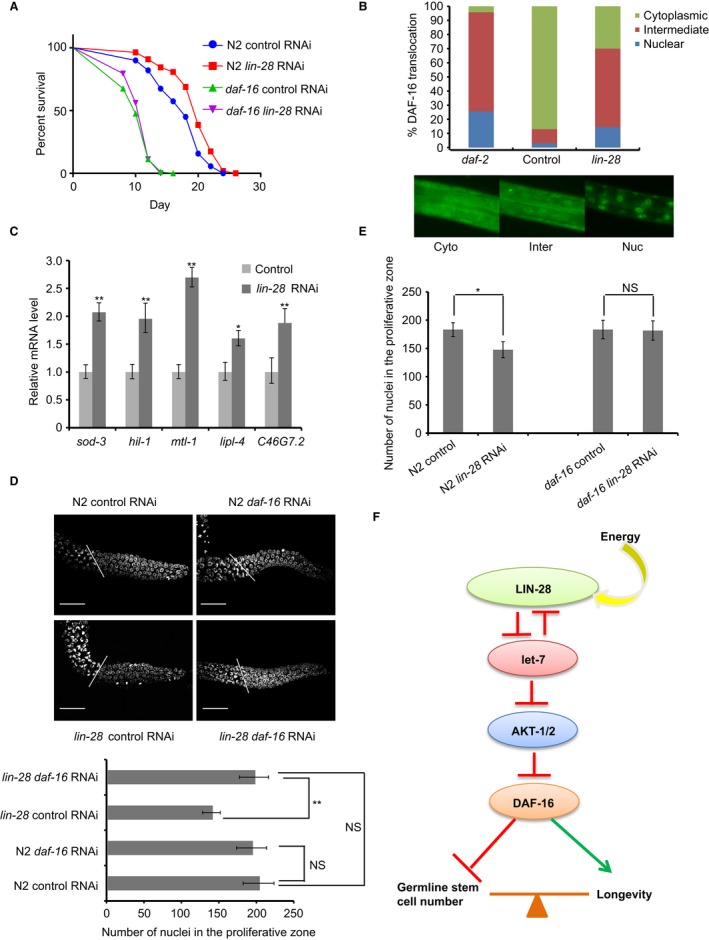
LIN‐28 requires DAF‐16 to influence germline stem cell number and longevity. (A) RNAi of *lin‐28* does not extend the lifespan of the *daf‐16*(*mu86*) mutant (log‐rank test *P* = 0.80). (B) RNAi of *lin‐28* increases *daf‐16::gfp*(*zls356*) translocation at early L3 stage. DAF‐16 translocation status is quantified in three categories manually as represented by the exemplary pictures with three biological replicates. (C) The mRNA levels of the classical DAF‐16 target genes *sod3*,* mtl‐1*,* hil‐1,* and *lipl‐4* increase upon *lin‐28* RNAi. Data represent mean ± SD (n = 3 biological repeats). *P < 0.05; **P < 0.0001. (D) Representative DAPI‐stained and average number of proliferative zone nuclei in wild‐type, *lin‐28*(*n719*) under control and *daf‐16* RNAi treatment. **P < 0.01 by two‐tailed Student's *t*‐test. NS, not significant. (E) RNAi of *lin‐28* reduces proliferative germ cell number of wild‐type worms significantly but does not change proliferative germ cell number of *daf‐16*(*mu86*) mutant worms. *P < 0.05 by two‐tailed Student's *t*‐test. NS, not significant. (F) Proposed model of germline LIN‐28 sensing the environmental energy level and acting through its downstream effectors let‐7, AKTs, and DAF‐16 to regulate reproduction and longevity by increasing the number of germline stem cells and repressing somatic maintenance. See also Fig. S5, Tables S1 and S2 (Supporting information).

To further confirm that *daf‐16* is downstream of *lin‐28*, we monitored the lifespan of *daf‐16*(*mu86*) null allele mutants fed with *lin‐28* RNAi bacteria. As expected, *lin‐28* knockdown could not extend the lifespan of *daf‐16*(*mu86*) null allele mutants (Fig. 6A, Table S1). To directly test *lin‐28*'s effect on DAF‐16 translocation, we performed *lin‐28* RNAi in the *TJ356* strain, which expresses the DAF‐16::GFP fusion protein. DAF‐16 translocation status was classified into three categories manually: (i) cytoplasm, (ii) intermediate, and (iii) nuclear. The percentage of worms in each category was then counted. The result showed that the percentage of nuclear and intermediate DAF‐16 in the *lin‐28* RNAi strain was significantly higher than the empty vector control, indicating that *lin‐28* knockdown stimulates DAF‐16 translocation at L3 stage (Fig. 6B). Consistent with enhanced DAF‐16 translocation, *lin‐28* RNAi‐upregulated DAF‐16 activated genes *sod‐3* and *lipl‐4* (Fig. 6C), while *lin‐28* overexpression repressed their expression (Fig. S5A). At the whole‐genome level, DAF‐16 targets are significantly enriched in the *lin‐28* RNAi‐upregulated genes according to our RNA‐seq data (GSEA FDR < 0.0001, Fig. S5B).

RNAi of *daf‐16* has been shown to rescue the reduced germline progenitor pool size in *daf‐2* larval (Michaelson *et al*., 2010). Similarly, we tested whether *daf‐16* RNAi can rescue the reduced germline cell number caused by loss of *lin‐28* and found that *daf‐16* RNAi itself hardly changed the number of germ cells in the distal proliferative zone, but it rescued the number of germ stem cells in *lin‐28*(*n719*) mutant to a nearly normal level (Fig. 6D, Table S2). Furthermore, *lin‐28* RNAi could not reduce germline stem cell number of *daf‐16*(*mu86*) mutant worms (Fig. 6E). This indicates that *lin‐28* acts through DAF‐16 to influence germline stem cell number and lifespan.

## Discussion

As a highly conserved RNA‐binding protein, the function of LIN‐28 has been intensively studied, including its regulation of growth and metabolism in stem cells, glucose metabolism, tissue repair, somatic reprogramming, and cancer. In this work, we show that in *C. elegans*, the essential developmental gene *lin‐28* oppositely modulates lifespan and germline stem/progenitor cell number through let‐7, AKT, and the downstream transcription factor DAF‐16, thus delineating the LIN‐28/let‐7/AKT/DAF‐16 axis as a program that plays an important role in balancing reproduction and somatic maintenance. Essential developmental genes that can regulate lifespan only do so in a modest way, such as *lin‐4*,* lin‐14* (15.8% extension) (Boehm & Slack, 2005), *sea‐2* (16.3% extension) (Huang & Zhang, 2011), *daf‐12* (9.8% extension in *daf‐12*(*rh273rh274*), 25.5% shortening in *daf‐12*(*rh61rh411*)) (Fisher & Lithgow, 2006), and *miR‐84;241* (no change compared to wild‐type but shorten the long lifespan of *glp‐1* mutant worms) (Shen *et al*., 2012). Likewise, we found that the extent of lifespan extension caused by *lin‐28* RNAi is also modest (< 30%). The strong evolutionary selections of these genes under developmental programs made homozygotes unmanageable for lifespan assays, or even shorten overall lifespan due to developmental defects. Such relatively weaker lifespan phenotypes, however, do make assays highly dependent on the reproducibility of the results. Therefore, all lifespan results in this study were repeated two or three times, and conclusions were all based on statistical significance.

As a heterochronic gene, loss of function in *lin‐28* accelerates differentiation of the hypodermal and vulva stem cells during L2 stage, and when *lin‐28* is overexpressed, it promotes self‐renewal and delays differentiation of the hypodermal and vulva stem cells, leading to the proliferation of hypodermal stem cells and cell cycle delay in vulva stem cells (Moss *et al*., 1997). We found that other than controlling stem cell differentiation of hypodermal and vulva cells during early development, LIN‐28 also promoted premature differentiation thus reduced the number of germline stem/progenitor cells at the larval stage in a germline‐autonomous manner. It has been reported that overexpression of LIN28A is associated with human germ cell tumors (Murray *et al*., 2013) and promotes primordial germ cell (PGC) development from embryonic stem cells both in vitro and in vivo in mice (West *et al*., 2009; Shinoda *et al*., 2013). Thus, the role of LIN‐28 in regulating stem cell proliferation/differentiation is conserved across species and tissues.

The trade‐off between reproduction and growth vs. somatic maintenance has been a central dogma of the ‘evolutionary optimization’ or the ‘disposable soma’ theory of aging, which assumes that the limited resources and energy availability to an organism or species necessitate a trade‐off in the allocation of resources and energy between somatic maintenance and other important physiological processes, mainly reproduction and growth (Kirkwood, 2005). Removing germline stem cells by germline laser ablation or *glp‐1*/NOTCH mutant can indeed increase the lifespan of *C. elegans* (Arantes‐Oliveira *et al*., 2002), supporting a trade‐off between germline progenitor pool size and longevity. However, the longevity phenotype of *lin‐28* knock down is unlikely due entirely to reduced germline stem cell number because of the following. First, a gene simultaneously regulating germline stem cell and lifespan does not necessarily mean that it has to regulate lifespan solely through germline defect. As an example, *rsks‐1* mutant reduces germline stem cell number and extends lifespan at the same time, but through different pathways (Korta *et al*., 2012). Second, *lin‐28* RNAi does not equal to germline removal, for example, induced by loss of *glp‐1*. Some phenotypes are even opposite between *lin‐28* RNAi and *glp‐1*:* glp‐1* mutant that has increased body fat, whereas *lin‐28* RNAi strain has decreased body fat; *glp‐1* mutant has normal body size, whereas *lin‐28* RNAi worms show smaller body size; *glp‐1* mutant is not known to mediate DR effect on germline stem cell and longevity, whereas *lin‐28* mediates DR effects on both germline stem cell and longevity. Furthermore, two of the let‐7 family members miR‐84 and miR‐241 were upregulated by germline loss in a DAF‐12‐dependent manner, where somatic DAF‐12 senses the absence of germline; mature let‐7 is also upregulated in *glp‐1* mutant, but in a DAF‐12‐independent manner (Shen *et al*., 2012). Here, we report the LIN‐28/let‐7 axis as a novel pathway paralleling the DAF‐12/miR‐84 and miR‐241 pathway to regulate longevity. In this pathway, although LIN‐28 itself mainly works in germline to regulate both germline stem cell meiotic entry and longevity, as a highly abundant circulating small RNA, let‐7 may circulate between germline and soma (Ohshima *et al*. 2010; Gandhi *et al*. 2013 He *et al*. 2015; Punga *et al*. ; Punga *et al*. 2016) . This spatiotemporal distribution of let‐7 is suggested to contribute to its cell nonautonomous regulation (Zhu *et al*., 2011).

DAF‐16 in germline, muscle, and epidermal cells has all been reported to regulate germline stem cell number (Michaelson *et al*., 2010; Qi *et al*., 2012). Intestine DAF‐16 is well known to regulate lifespan, including germline loss‐induced lifespan extension (Arantes‐Oliveira *et al*., 2002). We found that tissue‐specific knockdown of *daf‐16* only in the germline also reduces lifespan by 38%, but not as much as the 55% reduction by whole‐body knockdown (Fig. S5C), implicating that germline *lin‐28* may regulate DAF‐16 cell autonomously and cell nonautonomously (e.g., through cross‐tissue diffusion of let‐7) to regulate lifespan.

In mice, *let‐7* has been reported to target components of the insulin–PI3K pathway such as IGF1R, IRS2, and AKT to regulate glucose metabolism in mouse (Zhu *et al*., 2011). In *C. elegans*, DAF‐2, the sole receptor of insulin pathway, has no let‐7 binding sites and shows an additive effect with *lin‐28* in regulating lifespan, suggesting that *lin‐28* is independent of the insulin pathway although it shares the downstream PI3K pathway members in regulating longevity.

As an RNA‐binding protein, in addition to let‐7 precursors, LIN‐28 binds to a large number of mRNAs (Cho *et al*., 2012; Wilbert *et al*., 2012). To elucidate whether LIN‐28‐binding targets other than let‐7 are involved in LIN‐28‐regulated aging processes, we examined a *C. elegans* LIN‐28 CLIP‐seq dataset (Stefani *et al*., 2015) for the overlap of LIN‐28 induced differentially expressed genes and mRNAs directly bound by LIN‐28, and identified 80 overlapping genes. Among them, three genes, *acdh‐1*,* pat‐3,* and *ifg‐1,* are known to influence lifespan (Pan *et al*., 2007; Tacutu *et al*., 2012; De Haes *et al*., 2014). However, *ifg‐1* and *pat‐3* are targets of let‐7 as predicted by miRWIP (Hammell *et al*., 2008) and mRNA levels of *ifg‐1* and *acdh‐1* are significantly changed in *let‐7* mutant according to a microarray dataset (Hunter *et al*., 2013). Based on this, it is still unclear whether other *let‐7‐*independent LIN‐28 targets are involved in LIN‐28‐regulated aging processes.

In mice, Lin28 and let‐7 are reported to upregulate and downregulate insulin–PI3K–mTOR signaling pathway, respectively, which is known to promote aging (Zhu *et al*., 2011). One would expect that Lin28 promotes while let‐7 delays aging. Lin28 transgenic mice show enhanced tissue regeneration in mesenchyme and skin compartment by enhancing mitochondrial bioenergetics efficiency (Shyh‐Chang *et al*., 2013). Yet, excessive tissue regeneration has been shown to shorten lifespan in Pten^−/−^ mice (Yilmaz *et al*., 2006). In addition, Lin28 promotes PGC specification via let‐7 regulation of Blimp1 (West *et al*., 2009) and Lin28 deficiency compromises the size of germ cell pool (Shinoda *et al*., 2013). Only the precise dosage of LIN28/let‐7 that strikes optimal equilibrium between insulin signaling, tissue repair, and reproduction in different tissues would enhance mice longevity. Here, we used the model organism *C. elegans*, which lacks tissue repair process, and demonstrated the role of LIN‐28*/let‐7*/AKT1,2/DAF‐16 on simultaneously and oppositely regulating reproduction and longevity.

Finally, as all of the components of the LIN‐28/let‐7/AKT/DAF‐16 axis we depicted here are highly conserved from worms to human, we envision this pathway is very likely conserved in mammals, in balancing reproduction and lifespan under the influence of energy availability.

## Experimental procedures

### *C. elegans* strains, growth conditions, and RNAi

All *C. elegans* strains came from the Caenorhabditis Genetics Center: *lin‐28(n719), daf‐16(mu86), daf‐2(e1370), glp‐1(e2141ts), rrf‐1(pk1417), daf‐16::gfp(zls356), eat‐2(ad1116), akt‐1(ok525), akt‐2(ok393), let‐7(mg279)*,* ppw‐1(pk2505)*. The *lin‐28* overexpression strain *lin‐28*::gfp::*lin‐28* 3'UTR (ΔLCE) (*bpIs145*) was generated in a previous study (Huang & Zhang, 2011). All worms were cultured using standard techniques (Brenner, 1974) and *E. coli* OP50 as food except for RNAi. Mutants were all backcrossed to N2 at least six times to erase possible genetic background effects. All experiments were carried out at 20 °C except for the temperature sensitive *glp‐1* mutants, which were hatched at 20 °C and then shifted to 25 °C 12 h after hatching.

RNAi was performed on nematode growth media plates with 1 mm IPTG. All RNAi bacteria were from the Ahringer library and were sequenced before use. For all RNAi assays, the same HT115 bacteria carrying the empty L4440 construct were used as control. RNAi efficiency was assayed by qRT–PCR before each experiment.

### Lifespan and stress assays

Lifespan assay was conducted as described previously (Xue *et al*., 2007). Bleached embryos were hatched overnight on unseeded NGM plates; hatched, synchronized L1 larvae were then grown to the desired stage and transferred to normal or RNAi plates containing 20 μg mL^−1^ FUDR to prevent progeny growth. Worms were transferred every other day, and dead worms were recorded. Worms that crawled off were excluded from the experimental results. All experiments were independently repeated at least twice. The *P* value was calculated by log‐rank test on the Kaplan–Meier curves.

For stress response, worms were treated with stresses at adult day 4. For heat shock treatment, the plates were transferred to a 35 °C incubator and dead worms were picked out and recorded at 2 h intervals. For UV treatment, plates were exposed to 2000 KJ m^−2^ with the lids open, and then, survival rates were assayed every other day. For oxidative stress treatment, worms were immersed in 300 mm paraquat and transferred to a 20 °C incubator, and survival rates were assayed 12 h later.

### RNA isolation and quantitative PCR analysis

Total RNA was isolated from worms with TRIzol reagent (Invitrogen) at the stage described. One micrograms of total RNA was used to generate cDNA with superscript II reverse transcriptase (Invitrogen, Carlsbad, California, United States). qPCR analysis was carried out on Mx3000P machine (Stratagene) with SYBR Green regent (Takara, DALIAN, China). The *actin* gene was used for internal control, and the ΔΔCt method was used to calculate relative fold change. qPCR primers were listed in Table S3 (Supporting information).

### DAF‐16 translocation assay

For DAF‐16 translocation assays, 60 mid‐L3 staged worms were picked out and immediately mounted onto the slide with 0.1% sodium azide in S‐basal buffer. We visualized the nuclear translocation of DAF‐16 with a fluorescence microscope (Zeiss, Oberkochen, German) equipped with a digital camera. Worms were scored as having cytosolic localization, nuclear localization when localized clearly through the entire body or intermediate localization when nuclear localization is not complete. The number of worms exhibiting each level of nuclear translocation was recorded. The translocation experiment was repeated at least three times.

### RNA sequencing and data analysis

Total RNA of worms fed with empty vector, and *lin‐28* RNAi bacteria from L1 were isolated with TRIzol (Invitrogen) at adult day 4 stage and precipitated with isopropanol. RNA sequencing libraries were constructed following the Illumina RNA‐seq protocol with at least 10 μg total RNA. The quality of RNA sequencing data was assessed with Fastqc. Tophat was used to map the reads to the genome of *C. elegans* (ce10). Cufflinks was used to assembly the reads, and Cuffdiff (Trapnell *et al*., 2012) was used to search for the differentially expressed genes (FDR < 0.1). DAVID was used to find enriched GO terms among differentially expressed genes (Huang *et al*., 2009).

### Determination of number of nuclei in the proliferative zone, distance to transition zone, and mitotic index

The number of proliferative germ cells was performed as described (Pepper *et al*., 2003): Young adult worms were immobilized on a small plate using levamisole solution (0.1 mm). Gonads were dissected by cutting off heads at the pharynx with a 25 gauge syringe needle. Worms with extruded gonads were fixed with in cold methanol/formaldehyde for 5 min and washed twice with PBST. The worm pellet was then incubated with 4′6′‐diamidino‐2‐phenylindole solution (100 ng mL^−1^) for 5 min and washed twice with PBST. Worms were then transferred onto a 2% agarose pad and covered with a coverslip. Images were taken with the Zeiss Axio observer with a 630× zoom.

The distal edge of the transition zone border was defined as the first cell diameter in which two or more nuclei displayed the characteristic crescent shape. Distance to transition zone was measured in cell diameters from the distal tip to the transition zone border. The mitotic index is the percentage of metaphase and anaphase nuclei over the total number of nuclei in the proliferative zone.

### Western blot

Worms were grown synchronously to young adult stage and collected by washing with M9 buffer, packed by centrifugation at 3000 *g* for 1 min, and frozen in liquid nitrogen in ~200 μL loading buffer. Anti‐AKT‐1,2,3 (Sc‐8321; Santa Cruz, Dallas, Texas, United States) and anti‐tublin (Sigma, St.Louis, Missouri, United States) were used as primary antibodies.

## Author contributions

D.W. and J.‐D.J.H. conceived the study. D.W. and L.H. conducted lifespan analysis and RNA‐seq data analysis. D.W., M.S., and Y.Y. performed germline stem cell counting experiments. F.L. maintained all worm mutants. D.C. provided the evidence of the germline‐specific longevity effect of *daf‐16*. S.M., A.A., and H.Z. provided genetics and experimental design advices. H.Z. provided *lin‐28* overexpression worms. W.C. conducted digital imaging analysis. D.W. and J.‐D.J.H. analyzed the data and wrote the manuscript with help from C.D.G.

## Funding

No funding information provided.

## Conflict of interest

The authors declare no competing financial interests.

## Supporting information


**Fig. S1** LIN‐28 regulates body size and fat content of *C. elegans*, related to Fig. 1.
**Fig. S2** *lin‐28* expresses in the germline after adulthood and shares significant number of differentially expressed genes with *glp‐1*, related to Figs 2 and 3.
**Fig. S3** Mutation of *lin‐28*(*n719*) attenuates DR induced reduction in germline stem cell number and lifespan extension, related to Fig. 4.
**Fig. S4** DAF‐16 translocation is inhibited in *let‐7* mutant worms, related to Fig. 5.
**Fig. S5** *lin‐28* RNAi up‐regulated genes are enriched for DAF‐16 target genes and germline knockdown of daf‐16 reduces lifespan, related to Fig. 6.
**Table S1** Lifespan analysis results
**Table S2** Proliferative germ cell number
**Table S3** qRT‐PCR primers

**Table S4** Siginificantly expressed genes in *lin*‐28 RNAi worms compared
**Data S1** Experimental Procedures
