# Supplementary material for: LIN‐28 balances longevity and germline stem cell number in Caenorhabditis elegans through let‐7/AKT/DAF‐16 axis
Source: Aging Cell. 2016 Oct 11;16(1):113–24. doi: 10.1111/acel.12539 (PMC5242300; doi:10.1111/acel.12539)
Supplement: Supplementary file 1 — Fig. S1 LIN‐28 regulates body size and fat content of C. elegans, related to Fig. 1. Fig. S2 lin‐28 expresses in the germline after adulthood and shares significant number of differentially expressed genes with glp‐1, related to Figs 2 and 3. Fig. S3 Mutation of lin‐28(n719) attenuates DR induced reduction in germline stem cell number and lifespan extension, related to Fig. 4. Fig. S4 DAF‐16 translocation is inhibited in let‐7 mutant worms, related to Fig. 5. Fig. S5 lin‐28 RNAi up‐regulated genes are enriched for DAF‐16 target genes and germline knockdown of daf‐16 reduces lifespan, related to Fig. 6. Table S1 Lifespan analysis results Table S2 Proliferative germ cell number Table S3 qRT‐PCR primers [file ACEL-16-113-s001.pdf]

Fig. S1

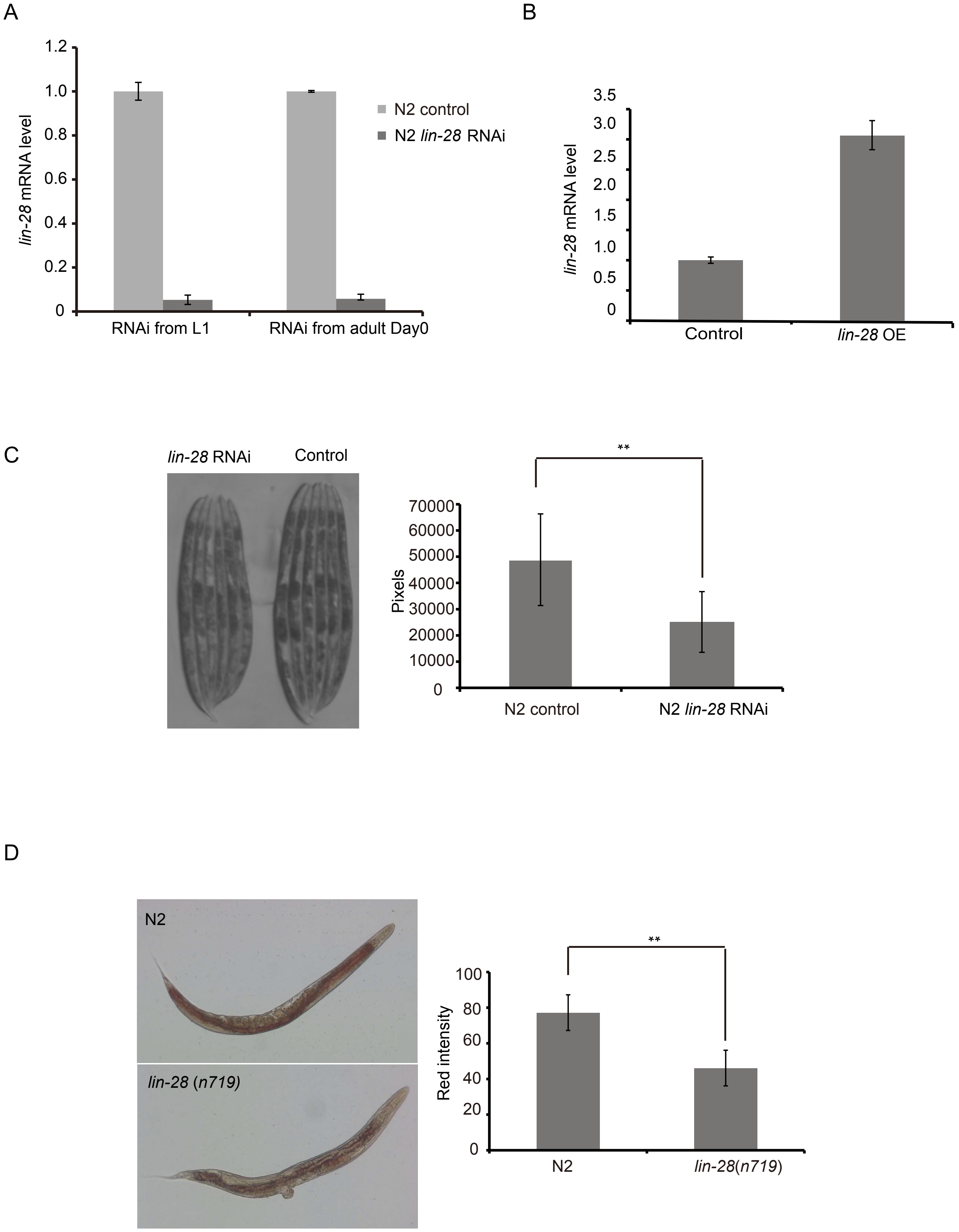
A
1.2
1.0
0.8
0.6
0.4
0.2
0
lin-28 mRNA level
N2 control
N2 lin-28 RNAi
RNAi from L1
RNAi from adult Day0
B
3.5
3.0
2.5
2.0
1.5
1.0
0.5
0
lin-28 mRNA level
Control
lin-28 OE
C
lin-28 RNAi
Control
**
70000
60000
50000
40000
30000
20000
10000
0
Pixels
N2 control
N2 lin-28 RNAi
D
N2
lin-28 (n719)
**
100
80
60
40
20
0
Red intensity
N2
lin-28(n719)

Fig. S2

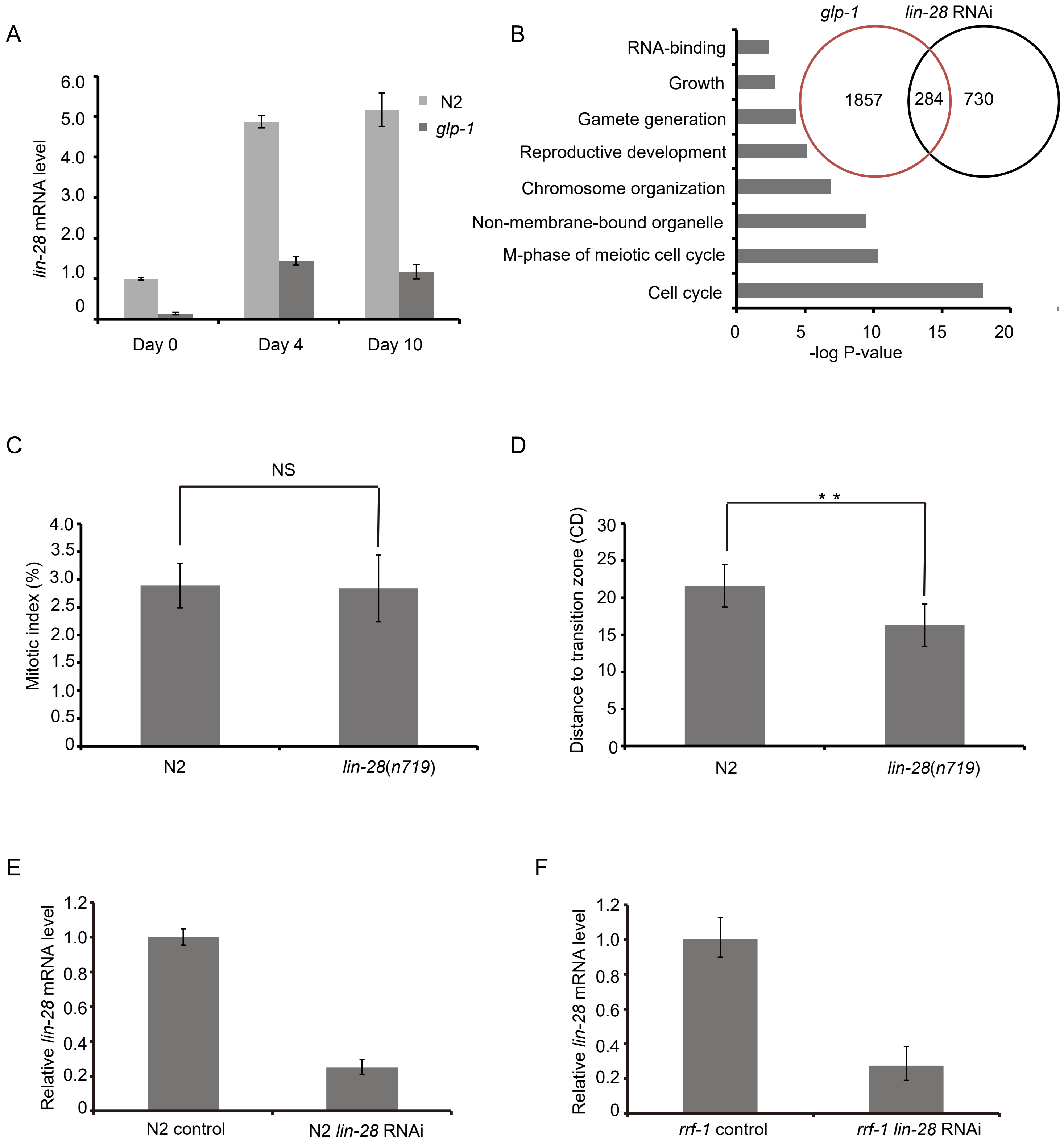
A
6.0
5.0
4.0
3.0
2.0
1.0
0
lin-28 mRNA level
N2
glp-1
Day 0
Day 4
Day 10
B
glp-1
lin-28 RNAi
1857
284
730
RNA-binding
Growth
Gamete generation
Reproductive development
Chromosome organization
Non-membrane-bound organelle
M-phase of meiotic cell cycle
Cell cycle
0
5
10
15
20
-log P-value
C
NS
4.0
3.5
3.0
2.5
2.0
1.5
1.0
0.5
0
Mitotic index (%)
N2
lin-28(n719)
D
* *
30
25
20
15
10
5
0
Distance to transition zone (CD)
N2
lin-28(n719)
E
1.2
1.0
0.8
0.6
0.4
0.2
0
Relative lin-28 mRNA level
N2 control
N2 lin-28 RNAi
F
1.2
1.0
0.8
0.6
0.4
0.2
0
Relative lin-28 mRNA level
rrf-1 control
rrf-1 lin-28 RNAi

Fig. S3

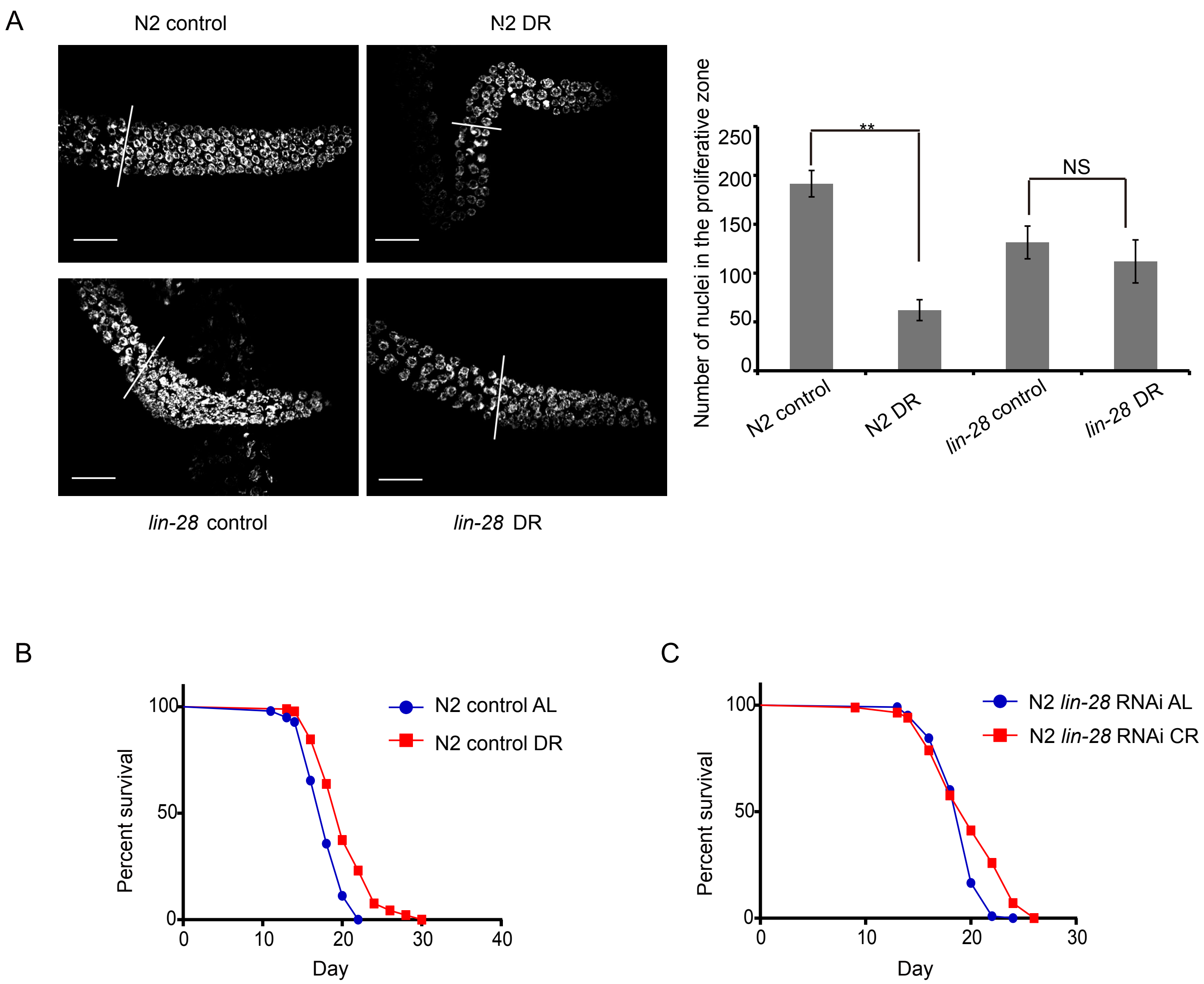
A
N2 control
N2 DR
lin-28 control
lin-28 DR
Number of nuclei in the proliferative zone
250
200
150
100
50
0
**
NS
N2 control
N2 DR
lin-28 control
lin-28 DR
B
Percent survival
100
50
0
0
10
20
30
40
Day
N2 control AL
N2 control DR
C
Percent survival
100
50
0
0
10
20
30
Day
N2 lin-28 RNAi AL
N2 lin-28 RNAi CR

Fig. S4

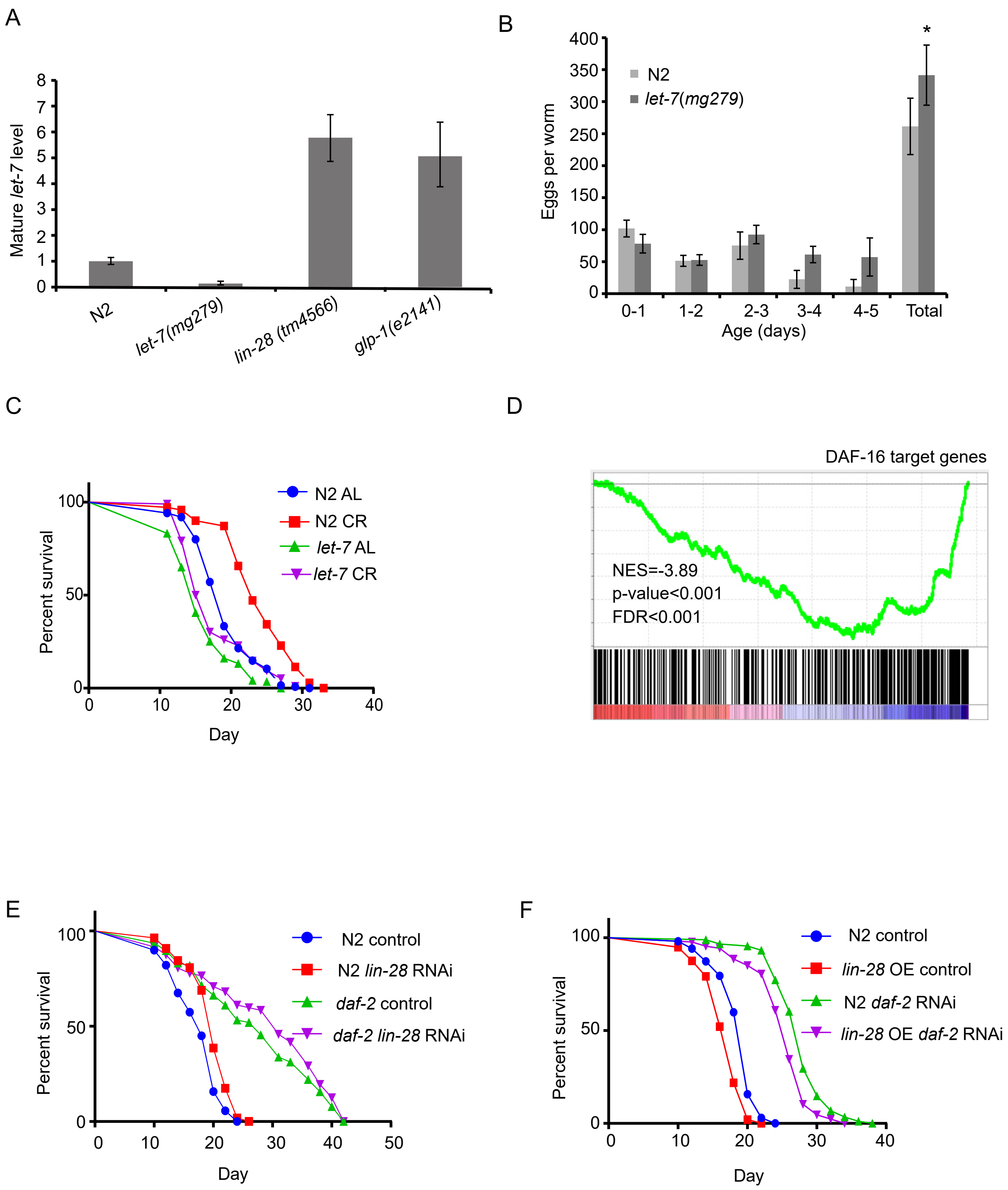
A
Mature let-7 level
0
1
2
3
4
5
6
7
8
N2
let-7(mg279)
lin-28 (tm4566)
glp-1(e2141)
B
Eggs per worm
0
50
100
150
200
250
300
350
400
N2
let-7(mg279)
*
0-1
1-2
2-3
3-4
4-5
Total
Age (days)
C
Percent survival
0
50
100
0
10
20
30
40
Day
N2 AL
N2 CR
let-7 AL
let-7 CR
D
DAF-16 target genes
NES=-3.89
p-value<0.001
FDR<0.001
E
Percent survival
0
50
100
0
10
20
30
40
50
Day
N2 control
N2 lin-28 RNAi
daf-2 control
daf-2 lin-28 RNAi
F
Percent survival
0
50
100
0
10
20
30
40
Day
N2 control
lin-28 OE control
N2 daf-2 RNAi
lin-28 OE daf-2 RNAi

Fig. S5

A

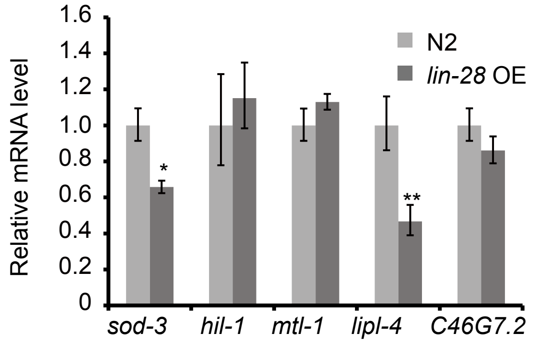
Relative mRNA level
1.6
1.4
1.2
1.0
0.8
0.6
0.4
0.2
0
N2
lin-28 OE
*
**
sod-3
hil-1
mtl-1
lipl-4
C46G7.2

B

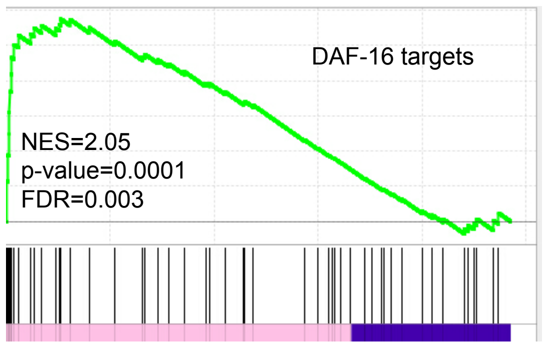
DAF-16 targets
NES=2.05
p-value=0.0001
FDR=0.003

C

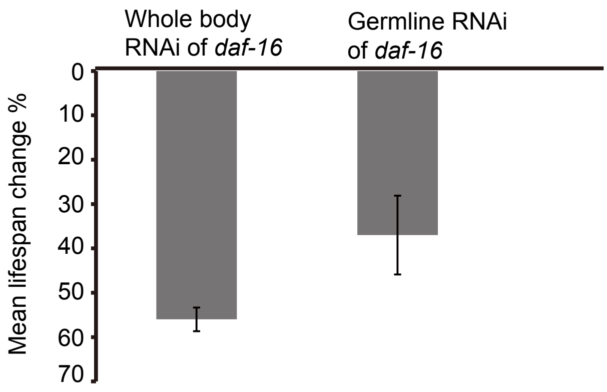
Whole body RNAi of daf-16
Germline RNAi of daf-16
Mean lifespan change %
0
10
20
30
40
50
60
70

Supplemental Figure Legends

Fig. S1. LIN-28 regulates body size and fat content of *C. elegans*, related to Fig. 1.

(A) RNAi efficiencies of *lin-28* RNAi from L1 stage or after adulthood are similar.

(B) *lin-28* mRNA shows a >3 fold increase in *lin-28* overexpressed worms.

(C) *lin-28* RNAi worms show smaller body size than that of wild-type ($p < 0.01$ by t-test).

(D) *lin-28* mutant worms show significantly lower fat content than wild-type worms by oil-red staining assay ($p < 0.001$ by t-test).

Fig. S2. *lin-28* expresses in the germline after adulthood and shares significant number of differentially expressed genes with *glp-1*, related to Fig.s 2 and 3.

(A) *lin-28* mRNA decreases dramatically in the germline-less mutant *glp-1(e2141)* after adulthood.

(B) On day4 of adulthood, differentially expressed genes caused by loss of *glp-1* and *lin-28* overlapped significantly ($p = 1.0 \times 10^{-6}$ by Fisher's exact test). These overlapped genes are enriched for "cell cycle" function.

(C) Mitotic index in wild type and *lin-28(n719)* germline proliferative zones. NS, not significant.

(D) Average distance in cell diameters (CD) from the distal tip to the transition zone in wild type and *lin-28(n719)* mutant at early adult stage. ** $p<0.01$ by two-tailed Student's t-test.

(E) *lin-28* RNAi efficiency in wild type worms (78.7% reduction).

(F) *lin-28* RNAi efficiency in *rrf-1* mutant worms (75.6% reduction).

Fig. S3. Mutation of *lin-28*(*n719*) attenuates DR induced reduction in germline stem cell number and lifespan extension, related to Fig. 4.

(A) Number of proliferative zone nuclei in wild type and *lin-28*(*n719*) mutant worms grown under normal conditions (bacterial concentration 1×10^{10} cfu/mL) and with DR (bacterial concentration 1×10^{8} cfu/mL). ** $p < 0.001$.

(B) DR conducted by a 100X dilution of food extends average lifespan of wild type worms by 7.1% (log rank test $p<0.0001$).

(C) when *lin-28* is knocked down by RNAi, DR cannot extend lifespan (log rank test $p=0.5$).

Fig. S4. DAF-16 translocation is inhibited in *let-7* mutant worms, related to Fig. 5.

(A) Mature *let-7* level is reduced to 9% compared with wild-type worms in the *let-7*(*mg279*) mutant strain and increased dramatically in *lin-28*(*tm4566*) and *glp-1*(*e2141*) mutant worms.

(B) *let-7*(*mg279*) worms have more off springs than control worms ($p < 0.05$ by t-test).

(C) Loss of *let-7* partially blocks CR induced lifespan extension. CR extends lifespan of N2 by 19.2% (log rank test $p<0.0001$), while only extends lifespan of *let-7* mutant by 12.6% (log rank test $p<0.002$).

(D) DAF-16 targeted genes are enriched in down-regulated genes when *let-7*(*mg279*) mutant microarray gene expression profiles are compared with the profiles of the control N2 strain (fold change data derived from Hunter *et al.* 2013.) (GSEA FDR = 0.003).

(E) *lin-28* RNAi extends the lifespan of *daf-2*(*e1370*) mutant worms (log rank test $p < 0.0001$) to a

similar extent as that of N2.

(F) *daf-2* RNAi still extends the lifespan of worms overexpressing *lin-28* (log rank test $p < 0.0001$).

Fig. S5. *lin-28* RNAi up-regulated genes are enriched for DAF-16 target genes and germline knockdown of daf-16 reduces lifespan, related to Fig. 6.

(A) The mRNA levels of the classical DAF-16 target genes *sod3*, *mtl-1*, *hil-1* and *lipl-4* decrease upon *lin-28* overexpression. Data are obtained by qRT-PCR and represent mean ± std (n=3 biological repeats). *, $p < 0.05$; **, $p < 0.0001$.

(B) According to the GSEA analysis of our *lin-28* RNAi RNA-seq data, DAF-16 target genes tend to be up-regulated by *lin-28* RNAi (FDR<0.001), consistent with the observation that *lin-28* RNAi stimulates DAF-16 translocation.

(C) Whole body RNAi of *daf-16* reduces lifespan by 56%, while knock down of *daf-16* by RNAi only in the germline reduces lifespan by 37%.

Supplemental Tables

Table S1. Lifespan analysis results

Strain	Mean lifespan± SEM (Days)	Worm number	log rank p-value	Fig.
N2 control from adult	18.6±2.1	233		
lin-28 RNAi from adult	20.2±2.0	272	vs N2 control p=0.0016	Fig. 1A
N2 control from L1 stage	18.2±1.1	256		
lin-28 RNAi from L1 stage	21.9±0.8	312	vs N2 control p<0.0001	Fig. 1B
N2 control from L3 stage	17.7±0.6	272		
lin-28 RNAi from L3 stage	20.7±0.4	268	vs N2 control p<0.0001	Fig. 1C
N2	19.2±1.7	237		
lin-28 OE	15.9±1.8	273	vs N2 p<0.0001	Fig. 1D
N2 control	18.5±0.8	257		
N2 *lin-28* RNAi	19.5±1.1	314	vs N2 control p<0.0001	
glp-1 control	19.8±0.9	284		
glp-1 lin-28 RNAi	19.3±0.6	295	vs *glp-1* control p=0.44	Fig. 3C
N2 control	19.4±1.2	236		
N2 *lin-28* RNAi	21.2±1.4	225	vs N2 control p<0.0001	
rrf-1 control	18.4±0.8	210		
rrf-1 lin-28 RNAi	20.0±0.8	227	vs *rrf-1* control p<0.0001	Fig. 3D
N2 control	19.5±0.4*	229		
N2 *lin-28* RNAi	21.3±0.2*	220	Vs N2 control p<0.0001	
ppw-1 control	18.0±0.5*	186		
ppw-1 lin-28 RNAi	17.8±0.2*	191	vs *ppw-1* control p=0.50	Fig. 3E
N2 control	17.4±0.2*	176		
eat-2 control	25.6±0.4*	156	vs N2 control p<0.0001	
N2 *lin-28* RNAi	19.3±0.1*	160		
eat-2 lin-28 RNAi	24.7±0.7*	173	vs N2 *lin-28* RNAi p<0.0001	Fig. 4B
N2 control	17.7**	110		
N2 *lin-28* RNAi	18.9**	100	vs N2 control p<0.0001	
let-7 control	13.7±1.2*	139		
let-7 lin-28 RNAi	14.1±1.1*	113	vs *let-7* control p=0.33	Fig. 5B
N2 control	19.7**	97		
N2 *lin-28* RNAi	22.9**	111	vs N2 control p<0.0001	
akt-1 control	22.6±1.9*	171		
akt-1 lin-28 RNAi	21.7±1.2*	171	vs *akt-1* control p=0.42	

akt-2 control	23.1+0.9*	199		
akt-2 lin-28 RNAi	21.9±0.7*	197	vs *akt-2* control p=0.06	Fig. 5D
N2 control	19.1±0.3*	200		
N2 *daf-16* RNAi	11.8±0.8*	306	vs N2 control p<0.0001	
let-7 control	11.4±0.2*	185		
let-7 daf-16 RNAi	10.8±0.8	301	vs N2 *daf-16* RNAi p=0.29	Fig. 5G
N2 control	17.3±0.6*	146		
N2 *lin-28* RNAi	19.3±0.1*	169	vs N2 control p<0.0001	
daf-16 control	14.7±0.8	298		
daf-16 lin-28 RNAi	14.3±0.7	284	vs *daf-16* control p=0.80	Fig. 6A
N2 control AL	18.2±0.3*	200		
N2 control DR	19.5±0.6*	202	vs N2 control AL p<0.0001	Fig. S3B
N2 *lin-28* RNAi AL	20.8±0.5*	202		
N2 *lin-28* RNAi DR	20.9±1.3*	193	vs N2 *lin-28* RNAi AL p=0.50	Fig. S3C
N2 AL	19.3±0.3*	263		
N2 CR	23.0±0.1*	124	vs N2 AL p<0.0001	
let-7 AL	15.9±0.2*	258		
let-7 CR	17.9±0.2*	195	vs *let-7* AL p=0.002	Fig. S4C
N2 control	17.2**	89		
N2 *lin-28* RNAi	19.5**	109	vs N2 control p<0.0001	
daf-2 control	30.4±2.8*	177		
daf-2 lin-28 RNAi	32.2±3.6*	174	vs *daf-2* control p<0.001	Fig. S4E
N2 control	19.2±0.6*	207		
N2 *daf-2* RNAi	30.0±1.1*	191	vs N2 control p<0.0001	
lin-28 OE control	15.9±2.6*	173		
lin-28 OE *daf-2* RNAi	24.8±0.6*	169	vs *lin-28* OE control p<0.0001	Fig. S4F

Three independent lifespan experiments were performed and pooled together for mean lifespan calculation unless otherwise noted. p values were calculated by log-rank test.

* Two independent experiments were performed;

** One independent experiment was performed, which occurred only for two standard controls.

Data shown as mean with standard error of mean (SEM).

Table S2. Proliferative germ cell number

Strain	Mean number of nuclei	n	p value(t-test)	Fig.
N2	191.5±13.6	18		
lin-28(*n719*)	131.4±16.7	24	vs N2 p<0.001	Fig. 2C
N2 control	189.2±12.3	22		
N2 *lin-28* RNAi	147.6±17.8	27	vs N2 control p<0.001	
rrf-1 control	187.3±12.6	32		
rrf-1 lin-28 RNAi	145.2±10.3	30	vs *rrf-1* control p<0.01	Fig. 3A
N2 control	191.1±11.9	28		
N2 *lin-28* RNAi	140.5±12.7	36	vs N2 control p<0.001	
ppw-1 control	194.0±12.9	23		
ppw-1 lin-28 RNAi	207.4±18.3	25	vs *ppw-1* control p=0.13	Fig. 3B
N2 control	174.4±10.8	23		
eat-2 control	95.0±17.1	34	vs N2 control p<0.001	
N2 *lin-28* RNAi	135.6±16.9	25		
eat-2 lin-28 RNAi	106.5±8.7	38	vs N2 *lin-28* RNAi p<0.001	Fig. 4A
N2 control	191.5±13.5	23		
N2 DR	62.0±10.6	25	vs N2 control p<0.0001	
lin-28(n719) control	131.3±16.2	25		
lin-28(n719) DR	111.9±9.8	30	vs *lin-28(n719) control* p=0.0012	Fig. S3A
N2 control	183.3±12.3	26		
N2 *lin-28* RNAi	147.6±14.2	26	vs N2 control p<0.0001	
let-7 control	187.7±9.9	25		
let-7 lin-28 RNAi	187.7±17.1	26	vs *let*-7 control p=0.9	
akt-1 control	168.9±11.1	28		
akt-1 lin-28 RNAi	188.3±17.1	36	vs *akt-1* control p=0.13	
akt-2 control	193.6±15.7	35		
akt-2 lin-28 RNAi	188.1±12.8	32	vs *akt-2* control p=0.24	Fig. 5E
N2 control	203.0±13.6	32		
N2 *daf-16* RNAi	193.7±16.7	30	vs N2 control ns	
lin-28(n719) control	140.1±11.8	35	vs N2 control p<0.0001	
lin-28(n719) daf-16 RNAi	196.8±19.8	33	vs *lin-28(n719) daf-16 RNAi* p<0.0001	Fig. 6D
N2 control	183.3±12.3	26		
N2 *lin-28* RNAi	147.6±14.2	26	vs N2 control p<0.0001	
daf-16 control	183.4±16.5	30		

daf-16 lin-28 RNAi	181.6±17.1	27	vs *daf-16* control p=0.76	Fig. 6E

n: number of worms examined.

The numbers proliferative nuclei were counted at young adult stage.

ns, not significant.

Table S3. qRT-PCR primers

Gene	primers	sequence(5'-3')
let-7	*let-7*-RT	GTCGTATCCAGTGCAGGGTCCGAGGTATTCGCACT GGATACGACAACTAT
	let-7-f	GCGCGTGAGGTAGTAGGTTGT
U18	*U18*-f	GGCAGTGATGATCACAAATC
	U18-r	TGGCTCAGCCGGTTTTCTAT
lin-28	*lin-28*-f	CAATATGCAAGGATTTCGGAGTC
	lin-28-r	CCTTGTCCTTCTACTTCTCCTG
sod-3	*sod*-3-f	CTAAGGATGGTGGAGAACCTTCA
	sod-3-r	CGCGCTTAATAGTGTCCATCAG
hil-1	*hil-1*-f	GCTACATGGATATGATCAAGGGA
	hil-1-r	TTTTGAGGACACTGCGGAG
mtl-1	*mtl-1*-f	TGTGAATGCAGTGGAGACAAG
	mtl-1-r	GCTCTGCACAATGACAGTTTG
hsp-12.6	*hsp-12.6*-f	TGGAGTTGTCAATGTCCTCG
	hsp-12.6-r	GACTTCAATCTCTTTTGGGAGG
lipl-4	*lipl-4*-f	CAAAACAAGACCTGGAAGAAACG
	lipl-4-r	GCTTCCCTGAACGACTTGAGA
fat-7	*fat-7*-f	ATAGTGTGGCGTAACGTGGC
	fat-7-r	TAGAGAGCAAATGAGAAGACG
C46G7.2	*C46G7.2*-f	CCACGTTGGCTCCATCTCTAG
	C46G7.2-r	ACAATGCGTTGGGCTTCTCT
actin	*actin*-f	TGCGACATTGATATCCGTAAGG
	actin-r	AGAGGGAAGCGAGGATAGATC

Supplemental Experimental Procedures

Worm body size and fat content quantification

A Matlab-based customized program is used for the image based worm body size and fat content quantification from oil red stained worms. We first transformed the worm image from the RGB to gray image，and then determined the threshold to distinguish the approximate worm body region in each image by Otsu's method (OSTU *et al.*, 1979) .We did dilate and erode operations (Vandenboomgaard *et al.*, 1992) to fill small holes and gaps to get more complete and accurate worm body regions. Then the worm body size was quantified by the pixels covered by a worm. The fat content was quantified by the sum of red content of red pixels (have higher value in the red channel in comparison to the blue and green channels) within a worm body region. Then the red content is divided by each worm's body size to represent oil-red intensity.

GSEA analysis of DAF-16 targeted genes in *let-7* or *lin-28* knockdown condition

GSEA (Gene Set Enrichment Analysis) software (Subramanian *et al.*, 1992) was used to conduct GSEA analysis. DAF-16 target genes was defined by Seung *et al.* according to their DAF-16 ChIP-seq data. *lin-28* RNAi RNA-seq is described in the main text (Seung *et al.*, 2005).*let-7* mutant versus control differential gene expressions were from Shaun *et al.* according to their microarray data. All genes were ranked by $\log_2$(fold change) versus control (Shaun *et al.*, 2013).

Supplemental References

OSTU, N. A Threshold Selection Method from Gray-Level Histograms (1979). *IEEE Transactions on Systems, Man, and Cybernetics – TSMC* **9**.

Vandenboomgaard, R. & Vanbalen, R.(1992) Methods for Fast Morphological Image Transforms Using Bitmapped Binary Images. *Cvgip-Graph Model Im* **54**, 252-258.

Subramanian A, Tamayo P, Mootha VK, Mukherjee S, Ebert BL, Gillette MA, Paulovich A, Pomeroy SL, Golub TR, Lander ES and Mesirov JP.(2005). Gene set enrichment analysis: a knowledge-based approach for interpreting genome-wide expression profiles. Proc Natl Acad Sci U S A **102**, 15545-15550.

Oh SW, Mukhopadhyay A, Dixit BL, Raha T, Green MR and Tissenbaum HA. (2006). Identification of direct DAF-16 targets controlling longevity, metabolism and diapause by chromatin immunoprecipitation. Nat genetics **38**, 451-457.

Hunter SE, Finnegan EF, Zisoulis DG, Lovci MT, Melnik-Martinez KV, et al. (2013) Functional Genomic Analysis of the let-7 Regulatory Network in *Caenorhabditis elegans*. PLoS Genet 9(3): e1003353.
